# Biomechanical Risk Classification in Repetitive Lifting Using Multi-Sensor Electromyography Data, Revised National Institute for Occupational Safety and Health Lifting Equation, and Deep Learning

**DOI:** 10.3390/bios15020084

**Published:** 2025-02-01

**Authors:** Fatemeh Davoudi Kakhki, Hardik Vora, Armin Moghadam

**Affiliations:** 1 Machine Learning & Safety Analytics Lab, School of Engineering, Santa Clara University, Santa Clara, CA 95053, USA; hvora@scu.edu; 2 Department of General Engineering, Department of Electrical & Computer Engineering, Santa Clara University, Santa Clara, CA 95053, USA; 3 Department of Computer Science and Engineering, Santa Clara University, Santa Clara, CA 95053, USA; 4 Department of Technology, College of Engineering, San Jose State University, San Jose, CA 95112, USA; armin.moghadam@sjsu.edu

**Keywords:** biomechanical risks, repetitive lifting, deep learning, EMG sensors, occupational safety

## Abstract

Repetitive lifting tasks in occupational settings often result in shoulder injuries, impacting both health and productivity. Accurately assessing the biomechanical risk of these tasks remains a significant challenge in occupational ergonomics, particularly within manufacturing environments. Traditional assessment methods frequently rely on subjective reports and limited observations, which can introduce bias and yield incomplete evaluations. This study addresses these limitations by generating and utilizing a comprehensive dataset containing detailed time-series electromyography (EMG) data from 25 participants. Using high-precision wearable sensors, EMG data were collected from eight muscles as participants performed repetitive lifting tasks. For each task, the lifting index was calculated using the revised National Institute for Occupational Safety and Health (NIOSH) lifting equation (RNLE). Participants completed cycles of both low-risk and high-risk repetitive lifting tasks within a four-minute period, allowing for the assessment of muscle performance under realistic working conditions. This extensive dataset, comprising over 7 million data points sampled at approximately 1259 Hz, was leveraged to develop deep learning models to classify lifting risk. To provide actionable insights for practical occupational ergonomics and risk assessments, statistical features were extracted from the raw EMG data. Three deep learning models, Convolutional Neural Networks (CNNs), Multilayer Perceptron (MLP), and Long Short-Term Memory (LSTM), were employed to analyze the data and predict the occupational lifting risk level. The CNN model achieved the highest performance, with a precision of 98.92% and a recall of 98.57%, proving its effectiveness for real-time risk assessments. These findings underscore the importance of aligning model architectures with data characteristics to optimize risk management. By integrating wearable EMG sensors with deep learning models, this study enables precise, real-time, and dynamic risk assessments, significantly enhancing workplace safety protocols. This approach has the potential to improve safety planning and reduce the incidence and severity of work-related musculoskeletal disorders, ultimately promoting better health and safety outcomes across various occupational settings.

## 1. Introduction

Occupational ergonomic assessments are essential for identifying injury risks and determining the priority areas for ergonomic interventions [1]. Major injury risk areas in occupational safety and health are work-related musculoskeletal disorders (WMSDs), which are often caused by physically demanding tasks that cause biomechanical overload [2]. Around 27.8% of workplace injuries in the industrial sector are due to WMSDs [3,4]. Therefore, understanding the connection between particular occupational tasks and the development of WMSDs is crucial for creating effective prevention strategies and improving occupational health and safety [5].

Among many occupational tasks that can lead to biomechanical overload, repetitive lifting is a major contributor to WMSDs [6]. Manual repetitive lifting includes moving objects, where improper techniques and prolonged lifting activities can lead to excessive muscle fatigue, resulting in occupational injuries and negatively impacting workers’ productivity, safety, and well-being [7]. Considering the established link between biomechanical loads and occurrence of WMSDs, the main factors contributing to WMDSs include the intensity, repetition, and duration of occupational tasks [8,9,10,11]. Biomechanical risk estimation for occupational tasks that involve lifting can be conducted using the National Institute of Occupational Safety and Health (NIOSH) method, though the revised NIOSH lifting equation (RNLE) [10].

The RNLE is a foundational tool that offers a systematic method for identifying potential risk factors in an occupational lifting task by assessing the intensity, duration, and frequency of lifting plus the geometric characteristics of the loads [6,10,12]. Occupational safety and ergonomic experts face several challenges in assessing the risks associated with various tasks. Traditional methods, such as RNLE, require trained professionals to manually record data, making continuous and personalized monitoring impractical and expensive. Consequently, conclusions are often drawn from limited observations, which may not be generalizable [1].

Additionally, ergonomic assessments are often time-consuming, costly, and inconsistent, with about one-third containing errors, 13% of which are significant enough to invalidate the evaluation [13]. A significant challenge in occupational safety is the predominantly reactive nature of traditional safety strategies, which typically analyze historical data only after injuries have already occurred [14]. For instance, reviewing workers’ compensation claims adopts a reactive stance on workers’ safety [14,15]. This underscores the necessity of innovative data sources and advanced analytical methods that can predict and mitigate risks before incidents happen.

Emerging proactive approaches utilize predictive ergonomics to analyze worker–environment interactions [4]. This proactive approach has the potential to mitigate the incidence of injuries by integrating real-time data and predictive analytics. Such a shift allows for a transition from merely responding to past events to proactively managing safety. This proactive paradigm aims to prevent injuries and foster a safer working environment by addressing potential hazards before they result in harm.

Having access to diverse sources of data appropriate for biomechanical risk modeling in occupational settings, machine learning and deep learning modeling techniques could be significantly useful in deriving practical insights from data [14,15] and crafting proactive strategies to mitigate WMSDs [12]. These technologies excel at analyzing intricate datasets to forecast potential safety hazards, thereby providing significant advantages in managing biomechanical and ergonomic risks in occupational settings [12,14,15,16].

Wearable sensors, such as surface electromyography (EMG) and inertial measurement unit (IMU) sensors, have transformed data collection in numerous fields by offering real-time insights into physiological activities and monitoring muscle activities [17,18]. Wearable sensors are characterized by being lightweight, small, and wirelessly connected and do not interfere with the workers’ natural motor strategies while lifting heavy loads [19].

These sensors are highly effective in tracking muscle activity by measuring the electrical signals produced by skeletal muscles, which is essential for assessing muscle health and the nerve cells that control them [6,18]. The data gathered from EMG sensors play a crucial role in clinical and biomedical diagnostics, rehabilitation [20,21], sports science [22], and the advancement of robotic and prosthetic devices [23], thereby significantly expanding their applications in occupational safety and health [6]. Additionally, EMG signals provide detailed insights into muscle activity, making them highly effective for analyzing muscle fatigue [7,24,25].

EMG sensors, attached to skin, offer the most natural means to estimate the torque required for a movement [26] and therefore could be optimally useful in research that investigates biomechanical analysis of material handling tasks. While much of the research on biomechanical load has focused on the use of IMU sensors [12,18,27,28,29,30,31,32], a few studies have used EMG sensors in the context of occupational-related tasks.

A pilot study in textile manufacturing used three EMG sensors and statistical analysis to measure the muscle fatigue change in overhead task works [33]. Another study on lifting assistive interventions in patient handling used EMG to analyze muscle activation during various patient-lifting scenarios [34]. Another study in an occupational-relevant environment analyzed the fatigue induced during repetitive tasks such as material handling [35].

The majority of research has analyzed the results from multivariable signals through statistical analysis after data collection. Yet, there is limited evidence on the application of other processing methods, such as machine learning and deep learning techniques, for proper and efficient physiological health monitoring in workplaces [36], especially for biomechanical risk assessment purposes in occupational settings [37]. Therefore, the literature has emphasized the need for further research investigating the applicability of machine learning and deep learning in signal processing for physiological health monitoring and workers’ occupational health, safety, and ergonomics [36,38]. These methods have the potential to enhance real-time data analysis, providing more accurate and efficient assessments of ergonomic risks and health outcomes [36].

### Research Motivation and Contribution

Traditional methods like the RNLE are commonly used to estimate biomechanical risks in manual lifting tasks to prevent WMSDs. However, the RNLE has significant limitations in real-time application, accuracy, and precision, particularly for repetitive lifting tasks [39]. Its static nature fails to account for the dynamic changes in lifting conditions, potentially leading to the misclassification of risk level.

Additionally, recent studies using the RNLE for risk classification have primarily focused on the lower back and employed a minimal number of sensors, limiting the comprehensiveness of the data collected [40]. Small participant samples further affect the generalizability of these findings [1]. Furthermore, the reliance on a small set of features in machine learning and deep learning models results in lower classification accuracy [19,28,41].

A few recent studies have employed the RNLE methodology for biomechanical risk classification, all focusing on the lower back area. For instance, Snyder et al. [42] utilized data from six IMU sensors for lower back risk classification and achieved an accuracy of 90.6% with CNN modeling. Thomas et al. [23] used the NIOSH dataset on IMU data to develop machine learning models involving 10 participants, attaining F1 scores between 0.96 and 0.98 across various model tuning scenarios. Donisi et al. [10] applied logistic regression with a single IMU sensor on the back, obtaining accuracy rates of 80–85% in repetitive lifting risk classification.

Similarly, Varrecchia et al. [40] employed IMU data and artificial neural networks for risk classification, achieving accuracy rates of 70–96% across different risk levels and tasks. The analysis of muscle activity in human movement, particularly involving the upper limbs, has significant implications for both professional activities and rehabilitation efforts [43]. Therefore, there is a research need to understand these movements in the upper body, with a focus on occupational ergonomics, aiming at identifying and mitigating WMSDs to inform the development of ergonomic interventions and rehabilitation protocols.

A systematic review on the most appropriate modeling for biosignal data demonstrated the efficacy and usefulness of deep learning models due to their complex structures and ability to capture patterns inherent in biosignals [44]. However, most research has analyzed multivariable signals through post-collection statistical analysis, highlighting the gap and the need for real-time sensory data analysis [36].

Additionally, there is limited evidence on the application of advanced processing methods, such as deep learning techniques, which is essential for real-time ergonomic risk assessment [28,36]. The applicability and efficiency of deep learning models based on wearable sensor data for human activity recognition are yet to be fully addressed [45,46]. Deep learning models provide superior accuracy, flexibility, and real-time data processing capabilities [47,48]. These advantages are especially valuable in occupational ergonomics applications where real-time predictive modeling enhances risk assessment and intervention methods, ultimately improving workplace safety and minimizing injury risks.

The primary motivation behind this research was to address critical limitations in traditional biomechanical risk assessment methods, such as the revised National Lifting Equation (RNLE), particularly in repetitive lifting tasks within occupational settings. The current approaches often lack real-time adaptability and fail to capture the dynamic nature of lifting tasks, which can result in inaccurate or incomplete risk assessments. Additionally, previous studies have largely focused on lower back risks with limited sensor data, small participant samples, and restricted feature sets, which hindered the comprehensive assessment of musculoskeletal risks.

To bridge these gaps, this research introduced a novel approach by integrating high-fidelity EMG data with advanced deep learning models. This approach broadens the scope of risk analysis to include upper body muscles and utilizes real-time, feature-rich data to improve the precision and practicality of ergonomic assessments. The following are the main contributions of this study:

- Comprehensive upper body analysis: extends biomechanical risk analysis to include shoulders and neck muscles, which are often overlooked in conventional studies focused mainly on the lower back.
- High-fidelity sensor data collection: utilizes EMG data from eight upper body muscles of 25 participants, offering a complete and more generalizable dataset compared to studies with fewer sensors and smaller sample sizes.
- Integration of RNLE with EMG sensors: applies RNLE to calculate lifting indices in tandem with EMG sensor data, providing a more relevant and dynamic risk assessment methodology for workplace ergonomics.
- Feature-rich data extraction: employs twelve statistical features from EMG signals instead of raw data, enhancing the interpretability and practical application of the results for occupational ergonomics.
- High classification precision: demonstrates the effectiveness of deep learning classification models, establishing them as reliable tools for real-time occupational risk assessment.
- Advancing real-time biosignal analysis: fills a research gap by illustrating the applicability of deep learning for the real-time analysis of biosignals, which is essential for proactive occupational risk management.

The remainder of this paper is organized as follows: Section 2 describes the material and methods used in this study. Section 3 presents the results and discusses the findings in detail. Finally, Section 4 addresses the study conclusions, its limitations, and suggests directions for future research.

## 2. Materials and Methods

**Figure 1:**
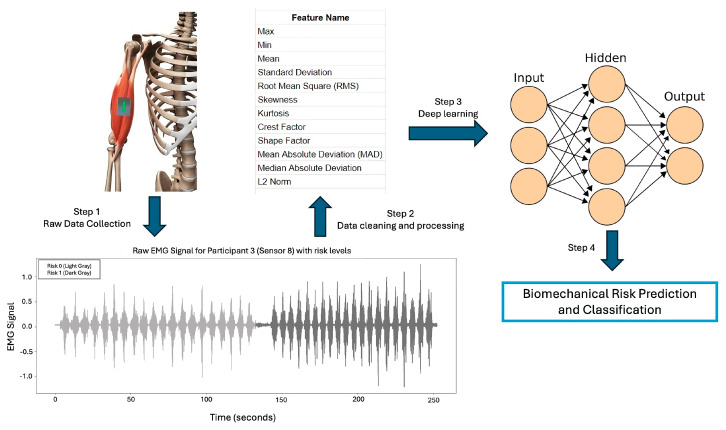
Overall methodology of this research included data collection from muscles, feature extraction from raw data, and using features to train deep learning models for biomechanical risk prediction on test data.

This section elaborates on the methodology and procedures used in this research. In this study, EMG signals were utilized to classify the risk levels associated with repetitive lifting based on the RNLE lifting index, which was calculated for designing the lifting tasks. The overview of the methodology is shown in Figure 1. We used signals that can be recorded in the workplace using wearable sensors, which are featured by being lightweight, small, and wirelessly connected [19]. These sensors do not interfere with the workers’ natural motor strategies while lifting heavy loads. The risk is categorized into two classes: no risk and risk. The methodology encompasses several critical steps: data preparation, feature extraction, and modeling using Convolutional Neural Networks (CNNs), Multilayer Perceptron (MLP), and Long Short-Term Memory (LSTM). Each participant was confirmed to be in good health, without any physical discomfort, medical conditions, or musculoskeletal disorders.

All study procedures in this research adhered to the Declaration of Helsinki and received approval from the Institutional Review Board and the Office of Research Compliance and Integrity of Santa Clara University (No: 23-12-2086). In addition, prior to their participation in this study, informed consent was obtained from all individuals.

### 2.1. Participants

This study involved a sample of twenty-five (*n* = 25) healthy college students who participated voluntarily and completed the experiments. The group consisted of 15 men and 10 women, with a mean age of 21.44 years, a median age of 19 years, and an age standard deviation of 4.98 years. The average weight of the participants was approximately 152.3 pounds (69.1 kg), with a standard deviation of 25.75 pounds (11.68 kg). The median weight was 155 pounds (70.3 kg). The average height of the participants was approximately 171.33 cm with a standard deviation of 11.75 cm, and the median height of 170.18 cm. All participants were healthy and did not have any prior medical conditions. None of the participants had any current or previous WMSDs.

### 2.2. Experimental Design Based on Revised NIOSH Lifting Equation

The recommended weight limit (RWL) in the revised NIOSH lifting equation (RNLE) is calculated as a function of several task variables and their respective multipliers, which are grounded in ergonomic principles detailed in the NIOSH Applications Manual [49]. The RNLE is a method designed to assess the biomechanical risks associated with manual handling tasks, specifically lifting activities. It computes the recommended weight limit (RWL), representing the maximum safe weight a healthy worker can lift. The RWL is determined using the formula

RWL = LC × HM × VM × DM × AM × FM × GM

where the load constant (LC) is the baseline weight, determined by the sex and age of the worker. LC represents the maximum weight that a healthy worker can lift under ideal conditions, including optimal posture, moderate frequency, and good coupling. For men under 45, it is 25 kg; for men over 45, it is 20 kg; for women under 45, it is 20 kg; and for women over 45, it is 15 kg. Each multiplier in the RWL formula modifies this constant to account for less-than-ideal task conditions. The multipliers are

- HM (horizontal multiplier): The HM is a factor that reflects the horizontal distance from the worker’s body to the load’s center of gravity. It is inversely proportional to the horizontal distance, with greater distances increasing risk. As horizontal distance increases, the biomechanical demand rises, reducing the HM and the RWL.
- VM (vertical multiplier): The VM is a factor reflecting the vertical height of the load relative to the floor. It is highest when the load is close to the worker’s waist height and decreases for higher or lower positions. The VM adjusts for the vertical location of the hands, with a height of 30 inches (knuckle height) being optimal. Deviations above or below this height reduce the VM.
- DM (distance multiplier): The DM is a factor based on the vertical travel distance of a load during lifting, with shorter travel distances being more favorable ergonomically. Larger distances reduce this multiplier, indicating increased risk.
- AM (asymmetric multiplier): The AM is a factor that accounts for asymmetry in the lifting task, such as twisting or uneven posture. It decreases as the asymmetry increases. The AM considers the angular deviation from the body’s mid-sagittal plane, where increased asymmetry reduces the multiplier due to the added biomechanical strain.
- FM (frequency multiplier): The FM is a factor that represents the frequency and duration of the lifting task. More frequent or prolonged lifting reduces this multiplier. The FM adjusts for the number of lifts per minute and the duration of lifting tasks, reducing the RWL as lifting frequency or duration increases.
- GM (grab multiplier): This factor reflects the quality of the grip on the load. Poor grip conditions result in a lower multiplier, increasing the overall risk. The CM evaluates the quality of hand-to-object coupling (good, fair, or poor) and its impact on grip and control, with poor coupling resulting in lower values.

All these multipliers are derived from rigorous studies detailed in the NIOSH Applications Manual [50], which provides equations, tables, and guidance for accurately measuring and calculating each component. By integrating these task-specific modifiers, the RNLE ensures a comprehensive assessment of the biomechanical and physiological demands of lifting tasks, allowing practitioners to identify and mitigate risks.

#### Lifting Index Calculation and Risk Classification

The RWL serves as a foundational metric for evaluating manual lifting tasks. Followed by calculating the RWL for each task, the lifting task risk should be determined. First, the lifting index (LI) is calculated. The lifting index (LI) is a critical measure in assessing the risk of a repetitive lifting task, as defined by the NIOSH guidelines. The LI is calculated using the formula

LI = Load Weight (L)/Recommended Weight Limit (RWL)

In the next step, the calculated LI values are compared to NIOSH-defined thresholds to classify the task as acceptable, increased risk, or high risk. Based on the calculated LI, the task is classified into one of three risk levels:

- LI ≤ 1.0: Acceptable risk. The task is considered safe for most workers.
- 1.0 < LI ≤ 3.0: Increased risk. The task may increase the likelihood of lifting injuries, and controls should be implemented to reduce the risk.
- LI > 3.0: High risk. The task exceeds the safe capabilities of most workers, and redesign is recommended to mitigate injury risk.

When the LI exceeds 1, it indicates a task with increased physical risk. This framework, as established in the NIOSH handbook, ensures that lifting tasks are evaluated systematically, making the RNLE a critical tool in ergonomic risk assessment and workplace safety planning [19]. In this study, we aimed for binary risk classification for LI ≤ 1.0 (as the low-risk lifting task) and 1.0 < LI ≤ 3.0 (as the high-risk lifting task).

**Figure 2:**
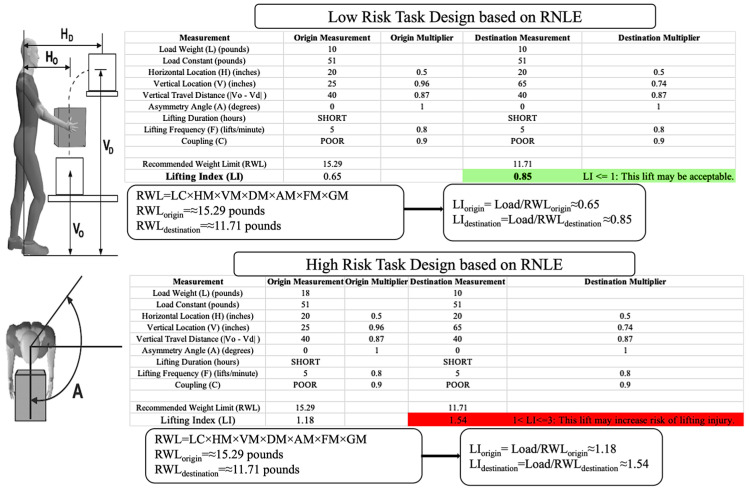
Detailed design and calculation of low-risk and high-risk lifting tasks using the RNLE. Green color represents the LI for low-risk task and the red color represents the LI for high-risk task.

The experimental design for this study is given in Figure 2, which provides comprehensive tables showing task measurements, calculated multipliers, and the resulting recommended weight limit (RWL) and lifting index (LI) for each lifting task. The primary objective of this study was to evaluate the biomechanical risks associated with two distinct lifting tasks:

1. Low-Risk Task: the lifting of a 10-pound (4.5 kg) box.
2. High-Risk Task: the lifting of an 18-pound (8.2 kg) box.

Both tasks required participants to lift the box from a height of 25 inches (63.5 cm) to 65 inches (165.1 cm), covering a vertical travel distance of 40 inches (101.6 cm). The horizontal distance from the body to the box was maintained at 20 inches (50.8 cm). The lifting frequency was set at 5 lifts per minute, with participants completing a total of 20 lifts per task. The coupling quality for both tasks was rated as Poor, reflecting suboptimal grip conditions.

The Revised NIOSH Lifting Equation (RNLE) formula was applied to calculate the RWL and LI for each task, with the smaller of the origin and destination RWL values used to determine the overall risk level. The smaller of the RWL and LI values should be used as the risk level for the entire task [40].

Based on the characteristics of this experiment (box, weight, coupling condition distances, etc.,), and according to NIOSH guidelines, the corresponding LI value in the study provides a direct measure of biomechanical risk:

- Low-Risk Task: The calculated LI was 0.85, indicating an acceptable level of risk.
- High-Risk Task: The calculated LI was 1.54, signifying a moderate risk level that may increase the likelihood of lifting injuries.

### 2.3. Experiment Tasks

This study comprised two trials, each designed to simulate manual handling activities with varying levels of physical exertion. In the first trial, participants lifted a 4.5 kg weight, consistent with low manual handling tasks. The task required lifting the weight from a height range of 61 cm to 152 cm at a frequency of 5 lifts per minute for a duration of 4 min, totaling 20 lifts. This setup aimed to mimic common workplace tasks involving nominal physical exertion. The lifting index for this task was calculated at LI = 0.85, indicating a nominal risk level and the lifting task being acceptable. To mitigate fatigue, participants were given a rest period of one minute between lifts, extendable to several minutes if needed.

The second trial mirrored the first in terms of procedure but introduced a higher weight of 8.2 kg for male participants and 7.2 kg for female participants, aligning with more strenuous workplace tasks. The same lifting height and frequency were maintained to facilitate a direct comparison of the increased physical demand with the first trial. The lifting index for this trial was LI = 1.54, reflecting a moderate risk level, which may increase the risk of lifting injury. As with the first trial, participants were provided a similar rest period to ensure adequate recovery and to maintain consistency in the experimental setup.

By maintaining consistent parameters across both trials, except for the weight lifted, we was able to effectively compare the physical demands and risk levels associated with low-risk versus moderate-risk manual handling tasks. This approach provided valuable insights into the impact of increased load on physical exertion and risk, contributing to a better understanding of manual handling in workplace settings.

### 2.4. Sensor Data Collection

**Figure 3:**
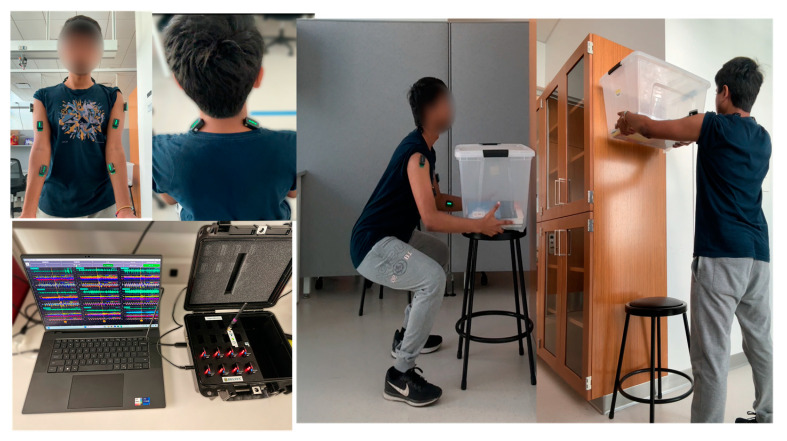
Data collection and experimental setup using DelSys sensor system.

The experiments were designed and performed under laboratory conditions. The details of the data collection process using DelSys sensor systems (Delsys Inc., Natick, MA, USA), highlighting the placement of sensors and the overall experimental setup, are shown in Figure 3. It shows the setup in which a participant was conducting the repetitive lifting task with eight EMG sensors attached to the upper body muscles: six sensors placed on the right and left arms and shoulders and two sensors on the neck.

Data were collected using DelSys Trigno EMG sensors (Delsys Inc., Natick, MA, USA), which employed four silver bar electrodes to detect EMG signals on the skin’s surface. The placement and data acquisition followed the guidelines detailed in the manufacturer’s instructions [51].

To ensure proper alignment, the sensors were positioned along the direction of muscle fibers and secured using the DelSys adhesive interface. Sensors were positioned along the direction of muscle fibers to ensure accurate and consistent signal acquisition. The sensors were attached to the following muscles:

- Medial deltoid muscles (2 sensors, one on each arm): positioned over the mid-belly of the muscle, oriented along the direction of the deltoid fibers. This placement captured shoulder abduction activity effectively.
- Bicep brachii muscles (2 sensors, one on each arm): placed on the midline of the biceps, avoiding tendon areas, to accurately measure muscle activity during elbow flexion.
- Levator scapulae muscles (2 sensors, one on each side of the neck): positioned on the posterior aspect of the neck along the direction of the muscle fibers to measure activity associated with scapular elevation and neck posture.
- Forearm flexor muscles (2 sensors, one on each forearm): attached to the mid-belly of the forearm flexors (such as the flexor carpi radialis), oriented along the muscle fibers, to monitor wrist and hand activity during task performance.

This standardized placement ensured repeatability and consistency in capturing the electromyographic signals for each task. The sensors were carefully aligned with the muscle fibers to minimize cross-talk and improve the reliability of the recorded data.

The EMG signals in this study were captured at a high sampling rate of 1259.26 Hz, enabling the precise monitoring of muscle activity. These signals encompass a frequency range of 20 to 450 Hz, critical for detecting muscle contractions. With a peak amplitude of 11 mV, the system provided accurate recordings, offering valuable insights into muscle fatigue, strain, and overall activity during various tasks.

### 2.5. Data Preprocessing

Electromyography (EMG) data were collected from eight muscles using surface sensors to monitor electrical activity during lifting tasks. The sensors sampled at an approximate frequency of 1259 Hz. Participants performed a series of 20 box lifts, using either a 10 lb (4.5 kg) or 18 lb (8.2 kg) box, over a 4 min period, averaging 5 lifts per minute. Rest intervals were provided to minimize fatigue. In total, 25 participants (P1–P25) contributed to the dataset, which comprised 7,061,086 data points, with an average of 282,443 data points per participant.

For each task, a CSV file was generated containing 11 columns: Participant, EMG_Time (timestamps), Sensor 1 to Sensor 8 (muscle activity), and Risk. The Risk column indicates the level of task risk, where ‘0’ corresponds to the low-risk 10 lb lift and ‘1’ to the high-risk 18 lb lift. Minimal preprocessing was required; only the header row specifying the sampling frequency was removed.

Participants were anonymized using unique identifiers, and the weight of the box was recorded in a dedicated column (’4.5’ for the 10 lb box and ’8.2’ for the 18 lb box). The time column provided accurate timestamps for each sensor reading, ensuring precise temporal alignment of muscle activity data. EMG signals from the eight muscles were stored in columns labeled “Sensor 1” through “Sensor 8,” corresponding to sensor placement on the body. To facilitate analysis, the dataset included a target variable labeled “Risk Level,” categorizing tasks as ’No Risk’ for the low-risk 4.5 kg lift and ’Risk’ for the high-risk 8.2 kg lift. This structured approach allowed for a clear distinction between the different levels of physical exertion and the associated risks. By organizing the dataset this way, we ensured the comprehensive tracking and categorization of the biomechanical data, which were crucial for subsequent modeling and analysis. For data preparation, we followed a similar approach as discussed in [52,53,54,55].

The first step was to perform normalization of the time series to ensure uniformity across the data. This step adjusted the values of the mean (μ) and standard deviation (σ) of the segments to approximately 0 and 1, respectively. The normalization formula is given by xi=(xi−μ)/σ, where xi represents the time-series value at position i, and xi is the normalized result. This normalization step was crucial for ensuring that the data were on a consistent scale, facilitating more effective feature extraction and modeling [56,57]. The next step involved segmenting the EMG signals into time windows, each containing 400 samples. To capture muscle activity effectively, we applied a sliding window technique for feature extraction. We empirically determined that a window size of 400 data points, approximately 0.32 s of EMG signal, was optimal for our analysis. Given that the participants’ lifting movements were brief, this window length was sufficient to capture relevant muscle activation patterns. Each window was moved forward by one data point, resulting in overlapping windows that preserved the temporal continuity and provided rich training data for machine learning models. This overlapping window approach ensured that minor variations in muscle activity were captured, thus improving the models’ ability to predict the risk level of each lift. The final dataset contained 7,061,011 entries after applying the sliding window, retaining the richness of the original data.

### 2.6. Feature Engineering

Feature selection is a crucial process in optimizing the performance of machine learning and deep learning algorithms, as an excessive number of features can lead to redundancy and increased model complexity [25]. By identifying the most relevant and distinctive features, we can enhance the modeling of sensor data and address challenges associated with high dimensionality [54,58]. While deep learning methods can automatically extract features and offer advantages in many scenarios, the features derived from traditional network structures and optimization algorithms can often be challenging to interpret, complicating the intuitive understanding of these models [59]. In this study, we employed a feature engineering approach to optimize data use for classification, which ensured more interpretable and meaningful features before developing deep learning models. Building on this methodology, statistical feature extraction from EMG signals has been widely used in prior studies to capture the characteristics of muscle activity in ergonomics and biomedical applications [7,12,27,30,45,60,61,62].

These features enhance the modeling process by summarizing the critical aspects of muscle behavior, facilitating the development of predictive models for risk classification. In repetitive lifting tasks, accurately identifying high-risk periods can lead to better intervention strategies, reducing the likelihood of musculoskeletal injuries and improving workplace safety.

For each window of EMG data, we computed 12 statistical features per sensor. The statistical features extracted, their formula, and description in the context of this research are presented in Table 1. The feature engineering process expanded the dataset to a total of 99 columns. For each of the 8 sensors, 12 statistical features were extracted, resulting in 96 columns. These were combined with 3 metadata columns (Participant ID, EMG_Time, and Risk) to complete the dataset. This comprehensive feature set effectively captured the complexity of muscle dynamics, which was essential for accurate risk classification.

Next, the dataset was divided into training and testing subsets using an 80–20 split ratio. To ensure a consistent distribution of low-risk and high-risk lifts across both subsets, stratified sampling was applied based on the Risk variable, minimizing potential model bias.

### 2.7. Classification Model Development

Feature selection is a crucial process in optimizing the performance of machine learning and deep learning algorithms, as an excessive number of features can lead to redundancy and increased model complexity [25]. By identifying the most relevant and distinctive features, we can enhance the modeling of sensor data and address the challenges associated with high dimensionality [52,58]. While deep learning methods can automatically extract features and offer advantages in many scenarios, the features derived from traditional network structures and optimization algorithms can often be challenging to interpret, complicating the intuitive understanding of these models [59]. Deep learning is revolutionizing the field of biosensing by facilitating the analysis of the large, complex, and longitudinal datasets produced by biosensors [44,59]. Deep learning models offer enhanced accuracy, adaptability, and the ability to process real-time data [47,48,63]. These are particularly beneficial in occupational ergonomics, where real-time predictive modeling can improve risk assessment and intervention strategies, thereby enhancing workplace safety and reducing injury risks. In developing binary classification models to assess high and low biomechanical risks in repetitive lifting tasks, we utilized Multilayer Perceptron (MLP), Convolutional Neural Networks (CNNs), and Long Short-Term Memory (LSTM).

The CNN architecture, modeled after the natural visual perception mechanisms of living organisms, excels with time-series signal analysis [64] and is effective at extracting significant patterns and features from complex and noisy biological data. Specifically, 1-dimensional CNNs (1D CNNs) have proven to be particularly valuable for biosensing applications [44]. MLPs are highly effective for classification modeling due to their flexible and robust structure, which enables them to accurately differentiate between classes [15]. The input layer of an MLP accepts the statistical features extracted from EMG signals, providing a solid foundation for analysis [44,50]. The hidden layers, comprising one or more fully connected layers with nonlinear activation functions such as ReLU, allow the model to capture the complex, nonlinear relationships within the data. The output layer, typically consisting of a single neuron with a sigmoid activation function, is well suited for binary classification tasks. LSTM networks belong to the recurrent neural network family of architectures and are adept at capturing dependencies within sequences, making them particularly effective for handling biosensor data, which are inherently sequential [44,65].

These models were chosen for their effectiveness in analyzing EMG data based on the statistical features extracted from EMG signals. These deep learning models are adept at capturing no-linear relationships within the EMG data, which is essential for accurately identifying risk patterns, making them particularly suitable for EMG data analysis. In this study, we developed three deep learning models with specific layers and parameters to optimize performance.

The MLP model starts with an input layer matching the shape of the training data, followed by three dense layers with ReLU activation functions, progressively reducing the number of neurons from 64 to 16, and ends with a dense layer using a sigmoid activation function for binary classification. The CNN model begins with an input layer for reshaped data, followed by a Conv1D layer with ReLU activation, a MaxPooling1D layer to reduce dimensionality, and a flattening layer to prepare the data for dense layers. It then includes two dense layers with ReLU activations and a final dense layer with a sigmoid activation for output. The LSTM model includes an input layer for reshaped data, an LSTM layer with 32 units to capture temporal dependencies, followed by two dense layers with ReLU and sigmoid activations, separately, for classification.

### 2.8. Model Performance Assessment

Based on the structure of the confusion matrix, various model performance metrics are derived to evaluate and interpret the efficiency of classification tasks. The confusion matrix elements, TPs (true positives), FPs (false positives), TNs (true negatives), and FNs (false negatives), form the basis for calculating these metrics. These are used to calculate various model performance measures. *Recall* measures the proportion of actual positive cases that are correctly identified by the model. A high recall indicates that the model effectively identifies most of the true positive instances, minimizing missed detections (false negatives). *Precision* represents the proportion of predicted positive cases that are actually positive. High precision implies that the model makes fewer false positive predictions, ensuring the predictions are reliable.

To evaluate and compare the performance of the proposed method with that of existing approaches, we employed the evaluation metrics commonly used in sensor-based human activity analysis studies [66], which are accuracy and F1 score. *Accuracy* indicates the proportion of correctly classified instances (both positive and negative) out of the total number of cases. While accuracy is a straightforward metric, it can be misleading if the dataset is imbalanced. The *F1 score* provides a harmonic mean of precision and recall, offering a balanced metric for evaluating model performance, particularly in cases where there is an uneven class distribution. We also used the *Matthews correlation coefficient* (MCC) for model evaluation. It is another measure of the quality of binary classifications. It returns a value between −1 and +1, where +1 indicates perfect prediction, 0 indicates no better than random prediction, and −1 indicates total disagreement between prediction and observation. MCC is robust against the proportion of different classes, making it a reliable metric for evaluating the robustness of a risk classification model. A high MCC value indicates that the model is likely to perform well across different datasets and scenarios.

-
$Recall=\frac{TP}{\left(TP+FN\right)}$

-
$Precision=\frac{TP}{\left(TP+FP\right)}$

-
$Accuracy=\frac{TP+TN}{\left(TP+FN+FP+TN\right)}$

-
$F1-Score=2\times \frac{\left(precision\times recall\right)}{\left(precision+recall\right)}$

-
$MCC=\frac{\left(TP\times TN\right)-\left(FP\times FN\right)}{\sqrt{\left(TP+FP\right)\left(TP+FN\right)\left(TN+FP\right)\left(TN+FN\right)}}$

## 3. Results and Discussion

Three deep learning models, Multilayer Perceptron (MLP), Convolutional Neural Network (CNN), and Long Short-Term Memory (LSTM), on the binary classification task of assessing repetitive lifting tasks into low- and high-risk groups, based on EMG sensor data from eight upper body muscles, are discussed here.

The classification was binary, distinguishing between high-risk and low-risk biomechanical loads that could lead to WMSDs. Using a stratified dataset to ensure a balanced representation of both risk categories, the model demonstrated high degrees of accuracy and reliability in its predictions.

Various model performance measures were calculated and are presented in Table 2.

### 3.1. CNN Model Performance

The CNN model stood out, with exceptionally high performance in classifying biomechanical risks (confusion matrix in Table 3). It achieves a precision of 98.92%, effectively identifying risks with minimal false positives, thereby avoiding unnecessary workplace disruptions and costs. The recall rate of 98.57% ensures that almost all risk conditions are detected, greatly enhancing workplace safety by minimizing the chances of missing potential hazards.

**Figure 4:**
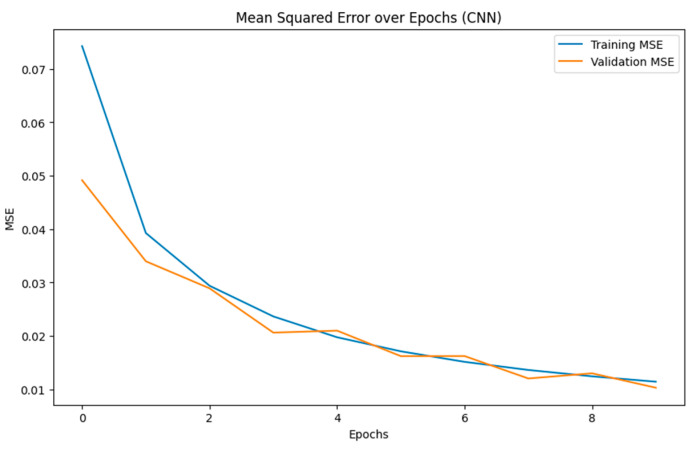
CNN model converging over 10 epochs.

The F_1_ score of 98.74% underscores the model’s superior balance between precision and recall, making it highly reliable in scenarios where both false positives and false negatives are critical. An overall accuracy of 98.66% reaffirms the CNN model’s effectiveness in risk prediction. The MCC of 97.32% indicates an excellent correlation between predicted and actual data, ensuring robustness and reliability across various case volumes and conditions. The CNN model achieved these results with a runtime of 20 min and convergence at only 10 epochs (Figure 4), demonstrating its efficiency in learning the spatial dependencies, which is crucial for accurate classification.

### 3.2. MLP Model Performance

The MLP model demonstrates robust performance in classifying biomechanical risks within occupational ergonomics (the confusion matrix is in Table 4). With a precision of 95.66%, it effectively minimizes false positives, thus preventing unnecessary interventions that could disrupt workplace operations and incur additional costs.

**Figure 5:**
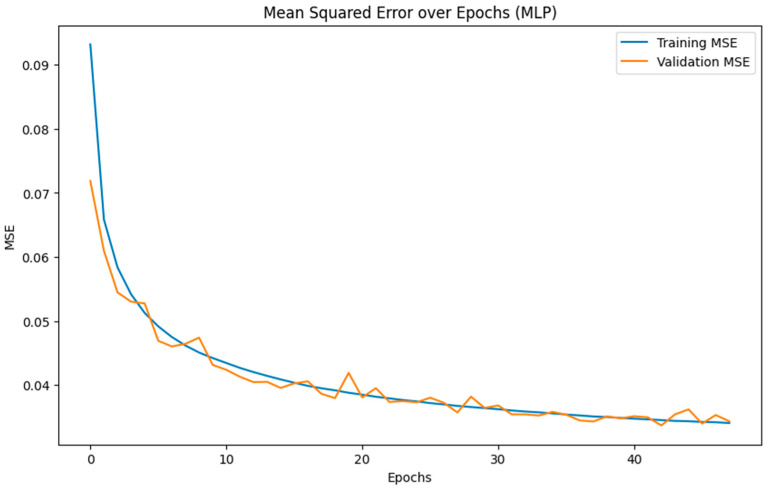
MLP model converging over 48 epochs.

The recall rate of 96.05% ensures that nearly all actual risk conditions are detected, enhancing workplace safety by reducing the likelihood of overlooking potential hazards. The F_1_ score of 95.86% highlights the model has well-balanced performance, which is important in scenarios where both false positives and false negatives can have significant consequences. Overall, the accuracy of 96.34% highlights the reliability of the MLP model in managing and accessing risk accurately. Furthermore, the MCC value of 96.16% indicates a strong correlation between predicted and actual data, reinforcing its quality and consistency across different classes and varied case volumes. The MLP achieved these results with a runtime of approximately 25 min and convergence at 48 epochs (Figure 5), showing efficiency in learning the relevant features from the data.

### 3.3. LSTM Model Performance

The LSTM model, while still effective, achieves lower metrics than the MLP and CNN models (confusion matrix in Table 5).

**Figure 6:**
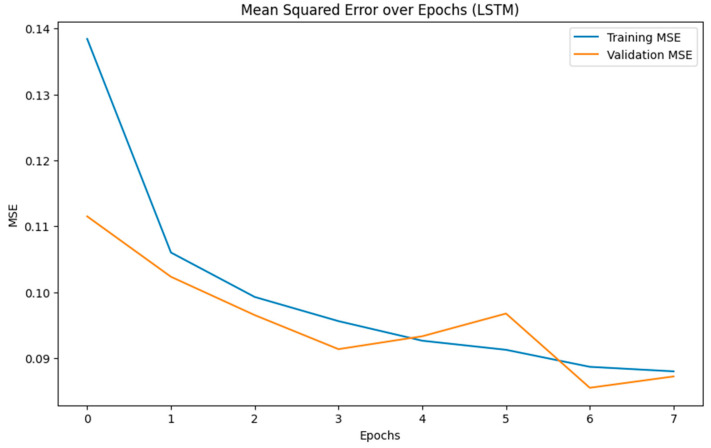
LSTM model converging over 9 epochs.

It achieves a precision of 89.65%, indicating a higher rate of false positives than the other models. The recall rate of 87.53% suggests that it detects most actual risk conditions but not as comprehensively as the MLP or CNN models. The F1 score of 88.58% reflects its balanced performance, though it is less optimal than the others. With an overall accuracy of 90.92%, the LSTM model proves to be less reliable in accurate risk prediction. The MCC of 85.36% highlights a weaker correlation between predicted and actual outcomes, suggesting potential limitations in diverse and dynamically changing workplace environments. Additionally, the LSTM model had a significantly longer runtime of 5 h, converging at nine epochs (Figure 6), indicating higher computational demands likely due to its recurrent nature.

### 3.4. Area Under the Curve

**Figure 7:**
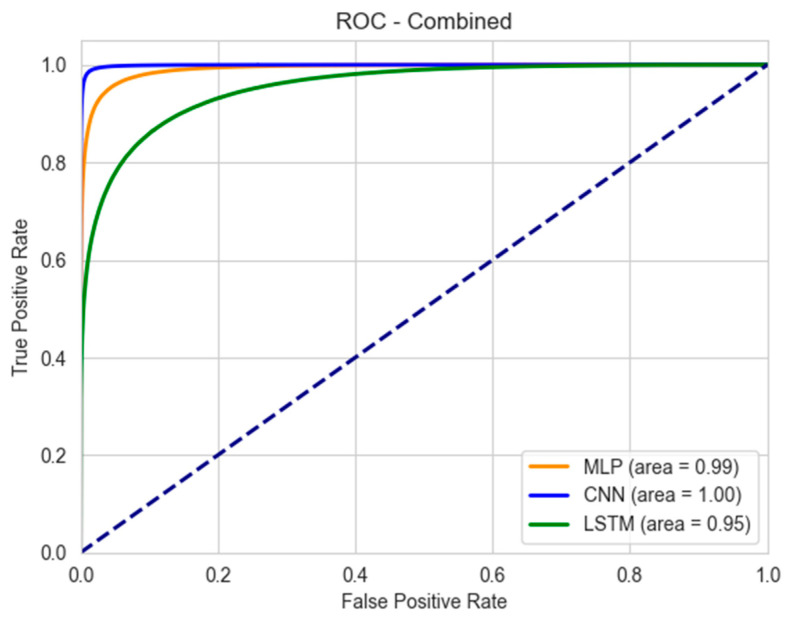
ROC-AUC for the three models. The dashed line represents a random classifier (AUC = 0.5), serving as the baseline for model performance.

We also analyzed the ROC-AUC values for the models, which are shown in Figure 7. The CNN model stands out with a perfect classification capability (AUC = 1), with no false positives or negatives. This performance ensures the accurate detection of high-risk tasks, thereby enabling timely interventions to prevent injuries. The MLP model also demonstrates high effectiveness, with an AUC of 0.99. This indicates its strong ability to distinguish between low- and high-risk tasks with minimal errors, making it a reliable tool for occupational safety assessments. Its performance ensures that most high-risk activities are accurately identified, supporting proactive measures to mitigate risks.

The LSTM model has a lower AUC of 0.95, suggesting it is less effective at classification compared to the CNN and MLP models. While still useful, its comparatively lower performance indicates a higher likelihood of misclassifying risk levels, which could impact the effectiveness of safety interventions.

The ROC curve visualization is particularly valuable in occupational settings where distinguishing between low- and high-risk classes is critical. The closer the curve to the left-hand border and then the top border of the ROC space, the better the model performance. The CNN–ROC curve closely hugs these borders, followed by the MLP model, while LSTM shows a more significant deviation.

### 3.5. Applications in Biomechanical Risk Modeling in Occupational Settings

The CNN model was found to serve as the most effective tool for the classification of biomechanical risks in occupational ergonomics based on EMG data, outperforming both the MLP and LSTM models in all key metrics. The ability of the CNN approach to capture local patterns through its convolutional layers makes it particularly powerful for tasks involving spatial data, such as EMG sensor data, which is pivotal for this classification task. Its superior performance, combined with efficient training time, underscores the practicality of using data with spatial dependencies for accurate risk prediction. The MLP model, while not as specialized as the CNN in handling spatial data, still performed commendably, with a good trade-off between accuracy and training time. This balance makes the MLP a viable option, especially in environments where computational resources are limited.

In contrast, the LSTM model design in handling sequential data did not offer an advantage for this specific task, as evidenced by its lower accuracy and significantly longer training time. These suggest that temporal patterns were either not as prevalent or critical in the EMG sensor data for distinguishing between risk levels. Alternatively, the LSTM might require further tuning or a different approach to better utilize the sequential nature of the data.

Overall, these results highlight the importance of choosing a model architecture that aligns with the nature of the data and the specific characteristics of the classification task at hand. The high performance of the CNN model validates its potential to significantly enhance preventative measures and safety protocols in occupational ergonomics, ultimately leading to safer working conditions and better health outcomes for employees.

The approach and results of this study have significant implications for improving biomechanical risk modeling in occupational settings, particularly when aligned with the revised NIOSH LIFTING EQUATION and the concept of the lifting index. By integrating high-precision wearable EMG sensors with deep learning models, such as CNN, MLP, and LSTM, this method enhances the real-time assessment of biomechanical risks during manual lifting tasks, a critical aspect of occupational safety and health. Traditional risk assessment methods often rely on historical data, which can be reactive and fail to address the real-time changes in biomechanical strain.

The use of wearable EMG sensors provides continuous, high-fidelity data on muscle activity, enabling a more dynamic and responsive risk assessment process. This real-time data collection allows for the immediate identification of high-risk lifting activities, facilitating timely interventions to prevent WMSDs. The revised NIOSH lifting equation is a well-established tool for evaluating lifting tasks, but it often depends on static factors and can overlook dynamic muscle activity and fatigue. By incorporating EMG data and machine learning models, the risk assessment process can be significantly refined. The precise measurement of muscle exertion and fatigue provides a more comprehensive evaluation of the lifting index, leading to better-informed decisions about task modifications, ergonomic interventions, and worker training programs.

The predictive capabilities of the CNN, MLP, and LSTM models enable the development of proactive safety interventions. These models can identify complex patterns and interactions in EMG data that traditional methods might miss, thus predicting potential injury risks before they occur. Implementing these predictive models supports the design of tailored interventions that can mitigate the risks associated with repetitive lifting tasks, reducing the incidence and severity of WMSDs.

Our findings suggest that integrating biosensor data with deep learning can significantly enhance the precision of ergonomic risk assessments. This methodological advancement supports the creation of more effective ergonomic protocols tailored to the specific needs of workers in various occupational settings. By providing objective and quantifiable measures of muscle activity, this approach ensures that risk assessments are based on empirical evidence, leading to more accurate and reliable safety protocols. Accurately assessing biomechanical risk and implementing effective interventions can lead to a substantial reduction in injury rates. The enhanced risk modeling approach proposed in this study contributes to safer working conditions by identifying and mitigating high-risk activities in real time. This offers the potential to improve the health and well-being of employees as well as the overall efficiency and productivity of material handling operations in manufacturing environments.

## 4. Conclusions

This paper presents an innovative and practical approach in biomechanical risk assessment that integrates high-fidelity EMG sensor data and deep learning models, specifically CNNs. Our approach demonstrated high precision in modeling and predicting the risk levels associated with repetitive lifting tasks. These results underscore the effectiveness of deep learning models in identifying risk levels based on EMG data, crucial for real-time and dynamic risk assessments in occupational settings.

Despite these promising outcomes, this study faces several limitations that need addressing in future research. First, the reliance on a controlled experimental setting with a small and homogeneous sample size of participants may limit the generalizability of the findings to broader, more diverse workforce populations. Additionally, the models were tested under specific lifting conditions; varying these conditions could affect model accuracy and applicability. Looking forward, this research opens several pathways for both academic exploration and practical applications. Future studies should aim to validate and refine these models across a wider range of occupational settings and with more diverse participant demographics to enhance the robustness and applicability of the risk assessment tools. Also, incorporating more varied data sources, such as environmental and psychosocial factors, could provide a more holistic view of the risk factors associated with occupational tasks. Advancing the capability for real-time data processing and feedback could transform these models into proactive systems that can dynamically predict and prevent high-risk situations during tasks. In addition, exploring the application of these models in other fields such as sports science, rehabilitation, and even non-industrial settings like healthcare could provide new insights and broaden the impact of this research.

In conclusion, while this study lays a robust foundation for using advanced analytical techniques in occupational risk assessment, the full potential of these technologies will be realized through continued innovation and broader application in diverse real-world scenarios. The proactive, predictive capabilities introduced here represent a significant step forward in occupational health and safety management, introducing promising solutions to protect workers and to enhance overall operational efficiency. By employing these insights, occupational safety professionals can make informed decisions on the most appropriate model to implement, ensuring high-risk tasks are accurately identified and effectively managed to enhance workplace safety.

## Figures and Tables

**Table 1:** Statistical features extracted from EMG signals and their description in the context of this research.

Feature	Formula	Description
Minimum	$\mathrm{Min}=\min\;(x_{i})$	Represents the lowest value in the window, identifying rest periods and ensuring muscles are not overstrained.
Maximum	$\mathrm{Max}=\max\;(x_{i})$	Represents the highest value in the window, indicating peak muscle activation and moments of high strain or exertion.
Mean	$\mu =\frac{1}{n}\;\overset{n}{\underset{i=i}{\sum }}x_{i}$	Represents the average value of the EMG signal, indicating the central tendency of muscle activity. Higher mean values suggest sustained contraction and possible muscle strain or fatigue.
Standard Deviation	$\sigma =\sqrt{\frac{1}{n}}\overset{n}{\underset{i=1}{\sum }}{\left(x_{i}-\mu \right)}^{2}$	Measures the variability in the EMG signal around its mean. Higher values indicate more fluctuation in muscle activation, often linked to inconsistent or strenuous muscle use.
Root Mean Square	$\mathrm{RMS}=\frac{\sqrt{\sum_{i=1}^{n}{x_{i}}^{2}}}{n}$	Provides the overall magnitude of the EMG signal. Higher RMS values correlate with greater muscle exertion, offering insight into activity levels.
Skewness	$\mathrm{skewness}=\frac{1}{n}\frac{\sum_{i=1}^{n}{\left(x_{i}-\mu \right)}^{3}}{\sigma^{3}}$	Captures the asymmetry of the EMG signal distribution. Values near zero indicate symmetry, while positive or negative skewness suggests bias toward higher or lower muscle activation.
Kurtosis	$\mathrm{kurtosis}=\frac{1}{n}\frac{\sum_{i=1}^{n}{\left(x_{i}-\mu \right)}^{4}}{\sigma^{4}}$	Reflects the peakedness of the EMG signal distribution. Higher values indicate sharp peaks, often corresponding to sudden bursts of muscle activity.
Crest Factor	$\mathrm{crest}=\frac{X_{max}}{XRMS}$	The ratio of the maximum value to RMS, highlighting sharp and brief muscle contractions relative to the overall signal magnitude.
Shape Factor	$\mathrm{shape}=\frac{RMS}{\mu }$	Ratio of RMS to the mean of absolute values, providing insights into the waveform shape and helping differentiate muscle activity patterns.
Mean Absolute Deviation	$\mathrm{MeanAD}=\frac{1}{n}\;\overset{n}{\underset{i=1}{\sum }}\left|x_{i}-\mu \right|$	Summarizes data dispersion around the mean. Higher values indicate greater variability in muscle activity.
Median Absolute Deviation	$\mathrm{MedianAD}=Median\;\left(\left|x_{i}-\tilde{x}\right|\right)$	A robust measure of variability, less sensitive to outliers, providing a stable assessment of muscle activity dispersion.
L2 Norm	$l2=\sqrt{\overset{n}{\underset{i=1}{\sum }}{\left|x_{i}\right|}^{2}}$	Represents the overall magnitude or energy of the EMG signal, useful for quantifying the intensity of muscle exertion.

**Table 2:** Model performance.

Metric	CNN Model	MLP Model	LSTM Model
Precision	98.92%	95.66%	89.65%
Recall	98.57%	96.05%	87.53%
F1 Score	98.74%	95.86%	88.58%
Accuracy	98.66%	96.34%	90.92%
MCC	97.32%	96.16%	85.36%

**Table 3:** Confusion matrix on test set, CNN model.

	Predicted: Low Risk	Predicted: High Risk
Actual: Low Risk	650,619	8142
Actual: High Risk	10,735	742,317

**Table 4:** Confusion matrix on test set, MLP model.

	Predicted: Low Risk	Predicted: High Risk
Actual: Low Risk	625,193	34,413
Actual: High Risk	29,192	723,120

**Table 5:** Confusion matrix on test set, LSTM model.

	Predicted: Low Risk	Predicted: High Risk
Actual: Low Risk	593,541	76,470
Actual: High Risk	93,927	639,625

## Data Availability

The data presented in this study are only partially available upon request from the corresponding author due to the privacy protocols as in the IRB approval documents.
